# Ovariectomy and Estradiol Supplementation Prevents Cyclophosphamide- and Doxorubicin-Induced Spatial Memory Impairment in Tumor-Bearing MMTV-PyVT Mice

**DOI:** 10.1523/ENEURO.0206-24.2024

**Published:** 2024-09-18

**Authors:** Robert Botelho, Rex M. Philpot

**Affiliations:** ^1^Departments of Psychiatry and Behavioral Neurosciences, Morsani College of Medicine, University of South Florida, Tampa, Florida 33613; ^2^Molecular Pharmacology and Physiology, Morsani College of Medicine, University of South Florida, Tampa, Florida 33613

## Abstract

Chemotherapy-related cognitive impairments (CRCIs) encompass cognitive deficits in memory, attention, and executive function that arise during and following chemotherapy. CRCI symptoms are predominantly reported by female cancer patients but also occur in males. These impairments may involve reduced estradiol levels, which then increases vulnerability to the impact of tumors and chemotherapy on cognition. This study utilized the MMTV-PyVT mouse model of breast cancer to test the hypothesis that impaired ovarian function and associated estradiol levels play a critical role in CRCI susceptibility. Mice were either ovariectomized (OVX) or underwent sham surgery. The OVX group then received supplemental estradiol (E^2^) *ad libitum* in the drinking water to maintain physiological hormone levels. After tumor development, mice were trained in the Morris water maze to assess spatial memory, and subsequently, they received weekly injections of either saline or a combination of cyclophosphamide (CYP; 66.7 mg/kg, i.v.) and doxorubicin (DOX; 6.7 mg/kg, i.v.) for 4 weeks. Spatial memory was reassessed 10 d and then 35 d, after the final injections. Results demonstrated a significant disruption of normal ovarian cycling in sham-operated mice treated with CYP + DOX, as well as significant spatial memory impairments when compared with OVX mice supplemented with E^2^. This study suggests that chemotherapy-induced ovarian damage and the consequent drop in circulating estrogens significantly contribute to vulnerability to CRCIs, underscoring the importance of estradiol in mitigating CRCI risks.

## Significance Statement

This study demonstrates that impairment of ovarian function in sham mice contributes to the manifestation of chemotherapy-related cognitive impairments (CRCIs). The observation that cognitive impairments were not present in E^2^-supplemented ovariectomized (OVX) animals suggests that disruptions of circulating E^2^ by chemotherapy mediate vulnerability to CRCIs. Unfortunately, OVX mice receiving E^2^ supplementation exhibited increased tumor volume in comparison with sham mice receiving tap water, suggesting that maintaining E^2^ can interfere with treatment. Therefore, E^2^ supplementation is unlikely to be an acceptable therapeutic intervention for CRCIs in cancers with tumors that express estrogen receptors. However, targeting mechanisms downstream of E^2^, such as high-affinity choline uptake, may be effective interventions for CRCIs.

## Introduction

Chemotherapy-related cognitive impairments (CRCIs) affect up to 75% of cancer patients receiving chemotherapy. CRCIs, which include deficits in spatial memory, attention, processing speed, and executive function, can persist for 20 years following treatment, significantly affecting patients’ quality of life (Koppelmans et al., 2013). Although CRCIs are observed in both men and women, most patients who report CRCIs are women (Janelsins et al., 2014), suggesting that women may be more vulnerable than men to the adverse cognitive effects of chemotherapy. Multiple mechanisms have been identified as contributors to CRCIs including direct central nervous system (CNS) damage from chemotherapeutic agents as well as indirect damage through induced neuroinflammation and oxidative stress (Fernandez et al., 2020). However, these mechanisms do not explain why women report CRCIs more frequently than men.

Many chemotherapeutic agents cause gonadal suppression or ablation, reducing levels of circulating sex hormones, and many women receiving chemotherapy exhibit amenorrhea and early menopause, resulting in decreased circulating estrogen levels (Walshe et al., 2006). It is well established that fluctuations in circulating estrogen levels affect the cognitive performance of females on a variety of behavioral tasks suggesting that damage to the ovarian follicles and resulting impairment in circulating estrogens may contribute to cognitive impairment (Rosenberg and Park, 2002). To this point, women experiencing amenorrhea following chemotherapy exhibit significant cognitive deficits when compared with chemotherapy patients who do not experience amenorrhea, implicating reductions of circulating estrogen in the manifestation of CRCIs (Walshe et al., 2006).

In support of findings from clinical studies, research in rodents has demonstrated that the chemotherapeutic agents cyclophosphamide (CYP; Cytoxan) and doxorubicin (DOX; Adriamycin) reduce the size and number of ovarian follicles (Mattison et al., 1981; Y. Wang et al., 2019). This is particularly relevant because CYP and DOX are two of the more commonly used chemotherapeutic agents and are often used in combination (AC chemotherapy) for the treatment of breast cancer. Weekly injections of subtherapeutic doses of CYP + DOX have been demonstrated to induce spatial memory deficits in female, but not male, Balb/C mice, suggesting greater vulnerability in females. Furthermore, research modeling CRCIs indicates that suppression of the estrus cycle in breast tumor-bearing MMTV-PyVT female mice using therapeutic doses of CYP + DOX is associated with poor spatial memory in the Morris water maze (MWM; Philpot et al., 2016).

The ovarian follicles are the primary source of circulating estrogens. Estrogens have several neuroprotective effects including anti-inflammatory actions in the CNS (Vegeto et al., 2008), inhibitory effects on microglia activation (Drew and Chavis, 2000; Vegeto et al., 2001), and inhibitory effects on the intracellular localization of NF-κB (Ghisletti et al., 2005), preventing downstream activation of inflammatory genes. Estrogen also increases glutamate uptake in astrocytes (Arevalo et al., 2010), preventing glutamate toxicity, and can act as an antioxidant (Razmara et al., 2007). Importantly, cancer patients exhibit increased levels of proinflammatory cytokines when compared with healthy controls (Rego et al., 2014), and several chemotherapeutic agents, including CYP and DOX, increase proinflammatory cytokines (Janelsins et al., 2012).

Thus, chemotherapy-induced ovarian suppression or ablation and the resulting reductions in circulating estradiol (E^2^) may render females uniquely vulnerable to chemotherapy-induced neurodegenerative processes that can promote neuronal injury (Ahles and Saykin, 2007).

The goal of the present study was to test the hypothesis that the adverse effects of CYP + DOX on spatial memory in female mice are dependent on vulnerability of the ovaries to ablation by chemotherapy. To this end, the spatial memory of ovariectomized (OVX) and sham-operated tumor-bearing female MMTV-PyVT mice was assessed prior to and following repeated CYP + DOX administration. Tumor-bearing mice were used because evidence suggests that the presence of tumors themselves may contribute to neurotoxicity and cognitive impairments (Denlinger et al., 2014; Olson and Marks, 2019). OVX mice were placed on E^2^-supplemented water to maintain physiological concentrations of E^2^ in the circulation. This manipulation allowed for systemic CYP + DOX to produce all adverse effects, known and unknown, with the exception of ovarian ablation and reducing circulating E^2^. The sham surgery group remained on tap water, rendering them vulnerable to chemotherapy-induced ovarian suppression or ablation and consequential reductions in circulating E^2^. Results indicate that spatial memory following the repeated administration of a therapeutic dose of CYP + DOX is protected when circulating E^2^ concentrations are maintained but is impaired when the ovaries are vulnerable to damage from chemotherapy, a consequence that reduces circulating E^2^.

## Materials and Methods

All procedures involving animals were performed in accordance with the animal care committee's regulations. Breeders and their offspring were given *ad libitum* access to water and standard Teklad Global 18% protein rodent chow (TD.2018, Envigo). *B6.FVB-Tg* (MMTV-PyVT) *634Mul/LellJ* mice (Jackson Laboratory) were bred in-house using hemizygous male mice and *C57BL/6J* female mice. This model was selected because it has a close similarity to human breast cancers requiring chemotherapeutic intervention (Guy et al., 1992; Maglione et al., 2001; Lin et al., 2003). Additionally, the *B6.FVB-Tg* strain is cross-bred with *C57Bl/6J* mice and was chosen over the *FVB-TgN* strain because the former exhibits slower tumor progression and lower metastasis rates than the latter, allowing for long-term studies of the effects of chemotherapy on cognitive function. Crucially, the *B6.FVB-Tg* strain is not vulnerable to the retinal degeneration and reduction of visual acuity that occurs in the *FVB-TgN* strain, making the former suitable for studies where intact vision is important.

### Genotyping

On postnatal day (P)21, when in-house–bred MMTV-PyVT mice were weaned, a tissue sample from the tail of each female mouse pup was collected and genotyped (Transnetyx) as MMTV-PyVT: Tg negative or positive. MMTV-PyVT: Tg-positive female mice (*N* = 53) were subsequently housed 3–4 per cage. MMTV-PyVT: Tg-negative females were used for other studies. Male mice were killed via CO^2^ inhalation followed by cervical dislocation.

### OVX

Female mice were randomly assigned to either OVX (*N* = 28) or sham surgery (*N* = 25) groups between P63 and P69 in young adulthood. Mice were then anesthetized with isoflurane (3–5%) in an induction chamber, removed, and placed on a rodent-specific nose cone apparatus with maintenance anesthesia (1–2%). Anesthetic depth was confirmed via toe pinch. Meloxicam (5 mg/kg, s.c.) and buprenorphine-sustained release (1.0 mg/kg, s.c.) were administered as multimodal analgesia at the time of surgery. Ancillary heat was provided using warm water circulation heating pads. The ovaries were removed via bilateral incisions (5 mm) midway between the base of the tail and the middle of the back. The location of the ovarian fat pad under the muscle was identified, and a small incision was made through the muscle. The ovaries were pulled through the incisions using blunt forceps by grasping the fat pad surrounding it, blood vessels were ligated, and the ovaries were removed. The muscle layer was then closed using absorbable sutures, with the skin closed via wound clips. For sham surgery mice, the same procedures were performed except for the ligation of blood vessels and removal of the ovaries. Following surgery, mice were individually housed to recover for 1 week. Meloxicam (5–10 mg/kg, s.c.) was given every 24 h for 48 h after surgery.

### Estradiol

Following surgery, OVX mice began estrogen replacement by supplementing the drinking water with 17β-estradiol (440 ng/ml). This method has been used to effectively maintain circulating estrogen at physiological levels (Gordon et al., 1986; Groeneveld et al., 2004). Water was monitored daily and changed regularly to verify normal fluid intake.

### Timeline, weights, tumor assessment, and measurement

**Figure 1.**
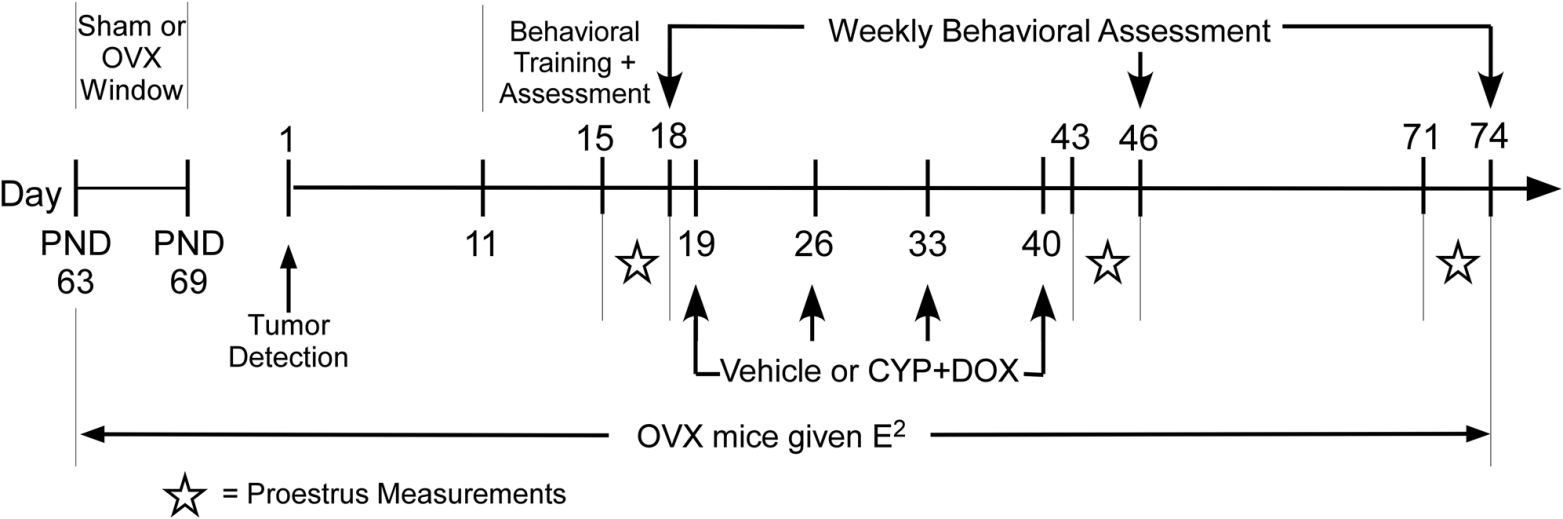
Study design and timeline for evaluating the impact of OVX and chemotherapy on spatial memory in MMTV-PyVT mice. This study began with 53 adult female MMTV-PyVT mice. Between P63 and P69, the mice underwent either sham surgery or OVX. OVX mice received estrogen (E^2^)-supplemented water postsurgery, while sham-operated mice had regular tap water. Mice were assessed weekly for tumor development, with Day 1 marking when tumors reached 50 mm^3^. Orientation to the MWM occurred on Days 11 and 12. Mice were trained from Days 13 to 17 to locate a consistently assigned hidden platform in one of the six zones. Proestrus status was checked on Days 15–18, 43–46, and 71–74. A baseline spatial memory test, including a probe trial, was conducted on Day 18. Mice were injected weekly on Days 19, 26, 33, and 40 with either a chemotherapy regimen (CYP and DOX) or saline. Reassessments of spatial memory in the MWM were conducted on Days 46 and 74 to gauge chemotherapy effects. The study concluded with 36 mice.

Mice were weighed and monitored weekly for tumor manifestation via manual manipulation and external tumor measurement using calipers. When a tumor >50 mm^3^ (using the modified ellipsoidal formula: volume_(tumor) _= ½(length × width^2^) was detected, the first day of that week was designated as Day 1 for that mouse (Fig. 1). Mice remained in the study unless a tumor ulcerated and reached a diameter >15 mm or a total volume of 2cm^3^ or other complications occurred. Mice were evaluated weekly for clinical signs requiring killing per Institutional Animal Care and Use Committee guidelines: ruffled coat hair, hunched posture, impaired mobility, cachexia (weight loss >20% of body weight), dehydration, anorexia, or dyspnea.

Starting with a tumor volume of 50 mm^3^ helps to ensure that all mice have tumors at a comparable stage of development and that the tumors are sufficiently established to mimic early-stage breast cancer. Importantly using this small tumor volume as a starting point allows sufficient time for the study of tumor-bearing controls who do not receive chemotherapy.

### Estrus cycle measurements

Assessment of proestrus status was conducted on Days 15–18, 43–46, and 71–74 using an Electronic Vaginal-Estrous Cycle-Monitor (Stoelting). Four-day windows for measurement were used because mice typically exhibit a 4 d cycle. The Electronic Vaginal-Estrous Cycle-Monitor is used to identify the different phases of the estrous cycle in rodents by measuring electrical resistance or impedance in the vagina. Elevated estrogen stimulates the proliferation of epithelial cells in the vagina, and impedance is higher (>3 kΩ) during proestrus, when the vaginal epithelium is thickest than during other phases (estrus, metestrus, diestrus). For the purposes of this study, a threshold of >4 kΩ was considered proestrus.

### Behavior

#### MWM

The MWM was chosen to assess cognitive function based on studies in mice indicating that chemotherapeutic agents impair performance in this task and findings in humans that spatial memory is impaired by chemotherapy (Hurria et al., 2006; Winocur et al., 2012, 2006; Philpot et al., 2016; Rendeiro et al., 2016).

*Apparatus*. A circular pool measuring 130 cm in diameter was filled with water at a temperature of 22–24°C. A clear Plexiglas-tiered platform, with a bottom-tier diameter of 15 cm and a top-tier diameter of 9.5 cm, was positioned in the water during all nonprobe trials. To visually obscure the platform, the water was made opaque with white nontoxic tempera paint (Tri Art). The pool was surrounded by a white polyvinyl curtain to block visual cues that might orient the subjects toward the experimenter or other external stimuli. Inside the curtain, distinct visual stimuli were placed to serve as static cues for spatial orientation. All behaviors were recorded by a video camera installed above the pool.

Behavior quantification within the pool utilized the Noldus EthoVision tracking software, which was used to define six equal-sized “virtual” zones within the pool. The software measured various parameters for each trial, including the average distance from the pool's perimeter, average swim velocity (VEL), total distance moved, latency to each zone, entries to each zone, latency to the platform, and time spent in the platform zone. The percentage of entries into the platform zone and the percentage of total distance moved (TDM) within the platform zone were subsequently calculated.

*Training and baseline assessment*. Beginning on Day 11, mice received orientation training consisting of five trials/day for a period of 2 d. This procedure has been demonstrated to improve the performance of female animals (Perrot-Sinal et al., 1996). For each trial, mice were placed in the pool facing the wall and allowed to swim to a visible platform. The starting location of the mice and the location of the platform were varied on each orientation trial, and the presence of the platform was indicated during orientation training by a Plexiglas rod (15 cm long, 3.8 cm diameter) that extended vertically from the platform 14.5 cm above of the surface of water. At the end of each trial, mice were towel dried and placed in a heated holding cage for 2 min before being returned to their home cages.

Spatial memory training consisted of five trials/day on Days 13–17. The intertrial interval was ∼25 min for each animal. During each trial, the platform was placed a few centimeter below the surface of the water. The platform location was varied across all six zones but remained constant for individual mice, so they could learn its location relative to cues in the environment. On every training day, each mouse was introduced into the pool from each of the five starting zones that did not contain the platform. The order of placement in each of the five zones was randomized for each animal. Animals were placed in the water, facing the wall, to start the trial. Each trial continued until the animal mounted the platform with all four paws or until 60 s elapsed. If a mouse failed to locate the platform within 60 s, the animal was gently guided to the platform. All mice were left on the platform for a minimum of 15 s before being removed from the water. On Day 18, Trials 1 and 2 occurred as described, with Trial 3 serving as a probe trial to assess spatial memory. During the probe trial, the platform was removed, and mice were allowed to swim for a total of 60 s before being removed from the apparatus. Following the probe trial, two additional platform trials were performed (Trials 4 and 5) to prevent possible extinction of spatial memory.

Following training and baseline assessment in the MWM (Day 18), mice that demonstrated spatial memory of the platform location by devoting ≥20% of their probe trial exploration to the zone that contained the platform during training were randomly assigned to saline or chemotherapy groups.

*Reassessment of spatial memory*. On Days 46 and 74, spatial memory was reassessed in the MWM to determine the effect of chemotherapy on spatial memory and the persistence of these effects, respectively. Mice received five trials on reassessment days with the platform for each individual mouse in the same location as during training on Trials 1, 2, 4, and 5. Trials 1 and 2 served to refamiliarize the mice with the MWM after a 28 d absence. To assess spatial memory, on Trial 3 (probe trial), we removed the platform from the pool, and we allowed the mouse to search for 60 s after being placed in a random, nonplatform zone. Trials 4 and 5 served to minimize extinction effects associated with the probe trial. The intertrial interval was ∼25 min for each animal.

### Chemicals and injections

Saline (0.9%) was made in-house using sterile ddH_2_O. CYP was obtained as a solid from Tocris Bioscience and dissolved in 0.9% saline to a concentration of 20 mg/ml. DOX (2 mg/ml) was obtained from Teva Parenteral Medicines. Based on group assignment at the baseline, mice received weekly injections of either CYP (66.7 mg/kg, i.v.) + DOX (6.7 mg/kg, i.v.) or equivalent volumes of saline on Days 19, 26, 33, and 40. To mimic clinical treatment, we administered CYP and DOX over four cycles, a common number of infusions for AC chemotherapy. The cumulative doses of CYP and DOX administered to mice correspond to 800 and 80 mg/m^2^, respectively, doses in the 4 week cumulative dose range for standard breast cancer chemotherapy in humans.

### Attrition

A total of 10 mice, six sham surgery and four OVX mice, did not demonstrate spatial memory of the platform location during the baseline probe trial and were removed from further study. Additionally, two sham mice receiving saline, three OVX mice receiving saline, and one OVX mouse receiving chemotherapy were removed during the study due to the tumor size or ulceration, and their data were not included in any analysis. Lastly, one mouse receiving chemotherapy was removed due to weight loss. At the end of the study, there were 8–10 mice per group and a final *N* of 36 for statistical analysis.

### Statistical analysis

Statistical analyses were performed using SPSS 27 (IBM). For all analyses, an a priori alpha of 0.05 was used to define statistical significance. In instances where using repeated-measure or mixed-factor ANOVA revealed a violation of sphericity, the Greenhouse–Geisser correction was used for all determinations of statistical significance. All post hoc tests were performed using Sidak's correction for multiple comparisons.

To assess whether surgery and chemotherapy altered the frequency of proestrus, *χ*^2^ analyses were performed on group proestrus frequencies obtained on Days 15–18, following four injections (Days 43–46), and at the end of the study (Days 71–74).

To assess weights, we performed a 2 (group) × 2 (treatment) × 10 (timepoint) mixed-factor ANOVA. To assess tumor number and tumor volume, we performed 2 (group) × 2 (treatment) × 12 (timepoint) mixed-factor ANOVA.

The VEL, latency to the platform (LTP), TDM, and platform zone entries (PZE) during the first platform trial on Day 18, after 4 weeks of chemotherapy (Day 46) and 5 weeks postchemotherapy (Day 74) were analyzed using 2 (group) × 2 (treatment) × 3 (timepoint) mixed-factor ANOVA.

To assess general activity, we analyzed TDM and VEL during the probe trial on Day 18, after 4 weeks of chemotherapy (Day 46) and 5 weeks postchemotherapy (Day 74) using 2 (group) × 2 (treatment) × 3 (timepoint) mixed-factor ANOVA.

To assess memory of the platform location, we analyzed the distance moved in the correct zone (DMCZ), entries into the correct zone (ECZ), time in the correct zone (TCZ), and latency to the correct zone (LCZ) during the probe trial on Day 18, after 4 weeks of chemotherapy (Day 46) and 5 weeks postchemotherapy (Day 74).

Partial correlations were performed to assess relationships between DMCZ, ECZ, TCZ, and LCZ to determine suitability for use in multivariate analysis (MANOVA) of spatial memory. Subsequently, a 2 (group) × 2 (treatment) × 3 (timepoint) MANOVA was conducted using moderately correlated measures (*r* = 0.2–0.7). According to Hair et al. (1998), moderate correlations (0.2 to 0.7) provide a good balance between having related variables that are not overly redundant, and MANOVA is most effective when the DVs are moderately correlated. This structure ensures that the covariance matrices are balanced and the assumption of homogeneity is more likely to be met.

To characterize MWM behavior on probe trials while controlling for individual differences in total activity, we analyzed the proportion of total movement devoted to each zone during the probe trials using a 2 (group) × 2 (treatment) × 3 (timepoint) × 6 (zone) mixed-factor ANOVA.

## Results

### Proestrus frequency analysis

The effect of surgery (sham/tap vs OVX/E^2^) and chemotherapy (saline vs CYP + DOX) on the frequency of proestrus on Days 15–18, 43–46, and 71–74 was assessed using *χ*^2^ analysis for independence. At baseline, following the OVX or sham surgeries, proestrus was observed in 89% of sham/tap mice and 91% of OVX/E^2^ mice and did not differ across groups. The observation of proestrus in OVX mice may be confusing; however, estrogens promote the proliferation of epithelial cells in the vagina and the Electronic Vaginal-Estrous Cycle-Monitor measures impedance associated with the thickness of the vaginal epithelium. Thus, this result indicates that the 17β-estradiol in the drinking water (440 ng/ml) of OVX/E^2^ mice maintained physiological levels of estrogen at concentrations sufficient to thicken the vaginal epithelium.

**Figure 2.**
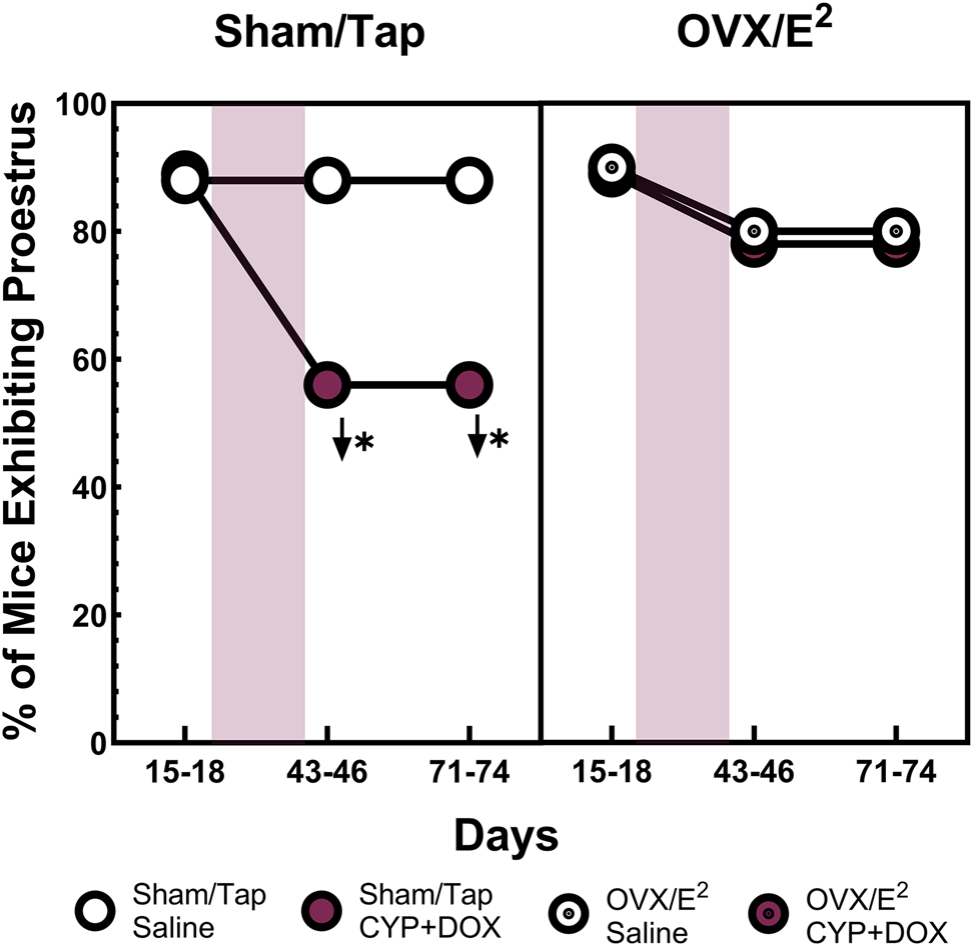
Changes in proestrus frequency across treatment phases. The percentage of sham-operated (sham/tap) and OVX/*E*^2^ mice exhibiting proestrus during three key phases: baseline (Days 15–18), after 4 weeks of chemotherapy (Days 43–46), and 5 weeks postchemotherapy (Days 71–74). Red shaded areas highlight the periods when mice received weekly injections of either chemotherapy agents (CYP and DOX) or saline. Significant within-group changes from the baseline are marked with arrows, and between-group differences at the same timepoints are indicated by asterisks. Statistical significance was determined by a *p* value of <0.05 following Sidak's correction for multiple comparisons.

The *χ*^2^ analysis revealed large (*ϕ* = 0.92) and significant [*X*^2^ (3, *N* = 36) = 30.44; *p* < 0.05] changes in the frequency of proestrus following four weekly injections of saline or CYP + DOX. On Days 43–46, 89% of saline-injected sham/tap mice exhibited proestrus, consistent with baseline measurements. Similarly, 80% of saline-injected OVX/E^2^ mice and 78% of CYP + DOX-injected OVX/E^2^ mice exhibited proestrus. However, only 56% of CYP + DOX-injected sham/tap mice exhibited proestrus over 4 d of measurement. This frequency was significantly lower than all other groups suggesting that CYP + DOX impaired normal estrus cycling in mice with intact ovaries (Fig. 2). When proestrus frequency was reassessed on Days 71–74, large and significant differences between groups remained [*ϕ* = 0.92; *X*^2^ (3, *N* = 36) = 30.4; *p* < 0.05], with saline-injected sham/tap mice, saline-injected OVX/E^2^ mice, and CYP + DOX-injected OVX/E^2^ mice all exhibiting proestrus frequencies between 78 and 89%, while only 56% of CYP + DOX-injected sham/tap mice exhibited proestrus, indicating a persistent impairment of estrus cycling in these mice.

### Tumor and weight changes

#### Weights

Factorial ANOVA demonstrated a large (partial *η*^2 ^= 0.125) and significant difference in group (sham/tap vs OVX/E^2^) weights over time (*F*_(9,288)_ = 4.554; *p* < 0.05). There was no significant difference in weights between groups at the start of the study (Table 1). However, 3 weeks after final injections (Day 64) and until the end of the study (Day 78), Sham/Tap mouse weights consistently increased at a slower rate (0.41–0.56 g/week) than for OVX/E^2^ mice (0.94–1.21 g/week), with OVX/E^2^ mice weighing significantly more by the end of the study.

**Table 1.** Weekly measurements of weights, tumor counts, and tumor volumes

Day	Sham/tap						OVX/E^2^					
	Saline			CYP + DOX			Saline			CYP + DOX		
	Weight (g)	Tumor		Weight (g)	Tumor		Weight (g)	Tumor		Weight (g)	Tumor	
		Number (#)	Volume (mm^3^)		Number (#)	Volume (mm^3^)		Number (#)	Volume (mm^3^)		Number (#)	Volume (mm^3^)
1	-	3.1 *±* *0.4*	73 *±* *11*	-	3.1 *±* *0.6*	66 *± 19*	-	3.5 *± 0.9*	73 *± 12*	-	3.0 *± 0.6*	105 *± 39*
8	-	3.3 *± 0.4*	125 *± 25*	-	3.8 *± 0.3*	119 *± 14*	-	4.1 *± 1.0*	191 *± 33*	-	3.9 *± 0.6*	286 *± 168*
15	21 *± 0.5*	4.8 *± 0.6*	310 *± 69*	20 *± 0.8*	4.6 *± 0.3*	257 *± 54*	21 *± 0.6*	4.8 *± 1.1*	366 *± 103*	22 *± 0.7*	4.8 *± 0.6*	466 *± 251*
22	21 *± 0.5*	5.6 *± 0.5*	577 *± 135*	20 *± 0.8*	4.7 *± 0.3*	201 *± 39*	21 *± 0.5*	5.7 *± 1.6*	529 *± 133*	22 *± 0.6*	4.9 *± 0.6*	482 *± 344*
29	21 *± 0.8*	6.1 *± 0.5*	883 *± 209*	20 *± 1.0*	4.4 *± 0.4*	108 *± 38*	21 *± 0.6*	6.2 *± 1.7*	830 *± 224*	21 *± 0.6*	4.7 *± 0.6*	361 *± 277*
36	21 *± 0.9*	6.8 *± 0.5*	1,150 *± 230*	20 *± 1.0*	3.4 *± 0.5*	73 *±* 33	21 *± 0.5*	6.8 *± 1.7*	1,167 *± 276*	21 *± 0.7*	4.8 *± 0.7*	398 *± 321*
43	22 *± 1.1*	6.9 *± 0.4*	1,503 *± 287*	20 *± 1.0*	3.7 *± 0.5*	87 *± 44*	22 *± 0.7*	7.4 *± 1.5*	1,824 *± 421*	20 *± 0.7*	4.8 *± 0.7*	320 *± 238*
50	22 *± 1.1*	7.4 *± 0.4*	2,071 *± 327*	21 *± 1.1*	4.4 *± 0.5*	141 *± 63*	22 *± 0.8*	7.8 *± 1.5*	2,620 *± 434*	21 *± 0.7*	4.9 *± 0.7*	453 *± 304*
57	22 *± 1.2*	7.4 *± 0.4*	2,997 *± 470*	21 *± 1.1*	5.1 *± 0.4*	261 *± 121*	23 *± 0.7*	8.5 *± 1.1*	3,740 *± 558*	22 *± 0.8*	5.0 *± 0.7*	887 *± 595*
64	23 *± 1.2*	7.6 *± 0.3*	2,971 *± 537*	21 *± 1.2*	5.3 *± 0.6*	470 *± 232*	24 *± 0.8*	8.3 *± 1.2*	4,721 *± 950*	23 *± 0.9*	5.7 *± 0.6*	1,044 *± 594*
71	23 *± 1.4*	7.7 *± 0.3*	3,374 *± 832*	22 *± 1.2*	5.8 *± 0.7*	901 *± 435*	26 *± 0.8*	8.3 *± 0.7*	7,037 *± 1,566*	24 *± 1.3*	5.9 *± 0.4*	1,346 *± 725*
78	24 *± 1.6*	7.6 *± 0.3*	4,190 *± 1,072*	22 *± 1.5*	6.4 *± 0.6*	1,208 *± 556*	27 *± 1.2*	8.1 *± 0.8*	8,878 *± 1,869*	25 *± 1.8*	6.7 *± 0.4*	1,998 *± 887*

Weekly recorded weights and tumor metrics, beginning from Day 1. Each entry shows the mean values accompanied by the SEM (in italic), along with the total number of tumors observed. The measurements are displayed for each experimental group, segmented by OVX status (sham or OVX) and treatment (saline or CYP + DOX). Data are aggregated from a final sample size of *N* = 36 mice.

#### Tumor number

Factorial ANOVA revealed large (partial *η*^2 ^= 0.347) and significant differences in tumor number changes over time due to treatment (*F*_(11,352)_ = 17.004; *p* < 0.05). From Day 8 to Day 43, there was a significant stepwise increase in the tumor number of saline-injected mice which subsequently plateaued. In contrast, mice in the CYP + DOX groups exhibited an increase in tumor number from Day 1 to 15 which then plateaued during chemotherapy from Days 22 to 43 and only increased significantly again on Day 78, demonstrating the effectiveness of CYP + DOX in suppressing tumor emergence.

#### Tumor volume

Factorial ANOVA revealed large (partial *η*^2 ^= 0.11) and significant changes in tumor volume (Table 1) over time due to interactions with the group and treatment (*F*_(11,352)_ = 3.954; *p* < 0.05). During the initial phase of the study, from Days 1 to 15 (the preinjection window), an increase in total tumor volume was exhibited in all animals, increasing by 344% (CI_95_ 311%, 379%). From Days 15 to 43, the tumors of saline-injected mice exhibited an increase in tumor volume of 393% (CI_95_ 369%, 419%). By contrast, from Days 15 to 43, the tumors of CYP + DOX-injected mice exhibited a 44% decrease (CI_95_ 35%, 51%). This change was consistent regardless of the animals’ OVX status, underscoring the effectiveness of CYP + DOX in reducing tumor volume. From Days 43 to 78, tumor volume in the saline-injected mice increased by 304% (CI_95_ 285%, 323%), while tumor volume changed by 687% (CI_95_ 587%, 807%) among mice previously receiving CYP + DOX during the same time span. Tumor volumes in the CYP + DOX group remained relatively smaller than those in the saline group by the end of the study (Day 78).

### MWM

#### Test day platform trials

Platform trials in the MWM should not be considered a measure of spatial memory specifically because the platform can be located without utilizing spatial cues if an effective search strategy is established (i.e., swimming in an expanding or reducing spiral or in concentric circles of increasing or decreasing diameters). However, performance on the first platform trial of each day can indicate group differences in retention of memories from prior sessions. Therefore, we examined group differences in VEL, LTP, TDM, and PZE on the first platform trial at the baseline (Day 18) and on reassessment Days 46 and 74 for indications of a disruption of long-term memory.

Analyses of Platform Trial 1 results on Days 18, 46, and 74 revealed a significant group by treatment interaction for LTP (partial *η*^2 ^= 0.128; *F*_(1,31)_ = 4.542; *p* < 0.05) and TDM (partial *η*^2 ^= 0.132; *F*_(1,31)_ = 4.72; *p* < 0.05) as well as a significant change across days of assessment for VEL (partial *η*^2 ^= 0.404; *F*_(2,62)_ = 20.99; *p* < 0.05), LTP (partial *η*^2 ^= 0.135; *F*_(2,62)_ = 4.83; *p* < 0.05), and TDM (partial *η*^2 ^= 0.100; *F*_(2,62)_ = 3.45; *p* < 0.05).

**Figure 3.**
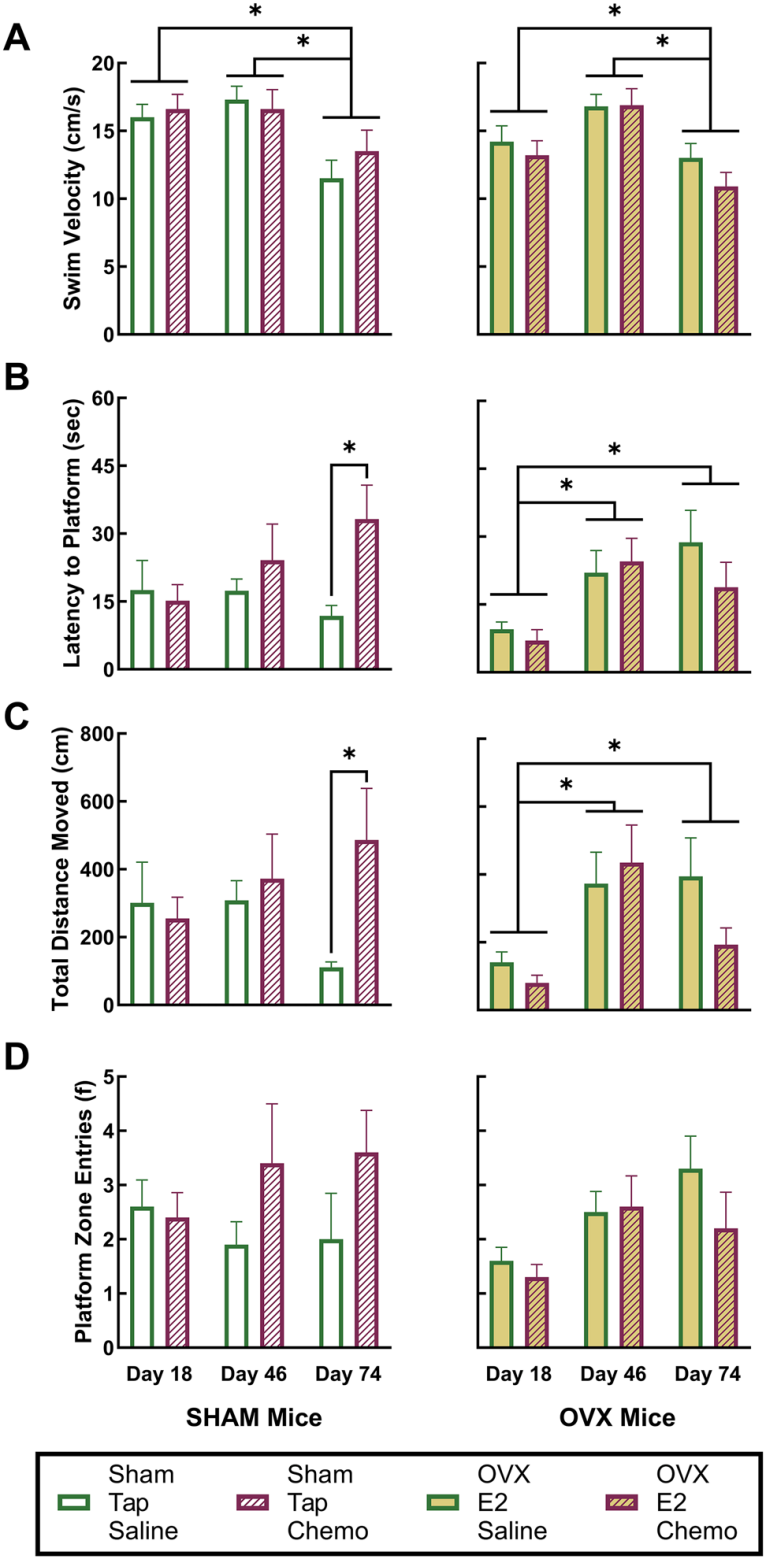
Behavioral metrics in Platform Trial 1. Panels ***A–D*** depict key performance metrics during the first platform trial in the MWM at three timepoints: baseline (Day 18), after 4 weeks of chemotherapy or saline injections (Day 46), and 5 weeks post-therapy (Day 74). The metrics include average VEL (***A***), latency to reach the platform (***B***), TDM(***C***), and the number of times mice entered the platform zone (***D***). Group means are presented with standard error of the mean (SEM). Symbols and color codes for different groups are detailed in the legend. Asterisks indicate statistically significant differences between groups, with significance set at a *p* < 0.05 following Sidak's correction for multiple comparisons.

Subsequent post hoc analyses using Sidak's correction for family-wise error revealed that VEL was significantly lower on Day 74 than on Days 18 or 46 (Fig. 3*A*). Because this effect did not differ between OVX/E^2^ mice and sham/tap mice or between mice receiving saline or chemotherapy, the effect likely reflects a tendency toward reduced activity as the mice mature and have more experience in the MWM.

Additional post hoc analyses revealed that, when compared with sham/tap mice receiving saline injections, sham/tap mice receiving chemotherapy took significantly longer and traveled significantly further to reach the platform on Day 74 Platform Trial 1, suggesting a chemotherapy-induced impairment. For OVX/E^2^ mice, post hoc analyses revealed that the Platform Trial 1 LTP (Fig. 3*B*) and TDM (Fig. 3*C*) was significantly lower at the baseline (Day 18) than observed in sham/tap mice; however, these differences were not present on Days 46 or 74. Additionally, the Platform Trial 1 LTP and TDM of OVX/E^2^ mice increased significantly from Day 18 to Days 46 or 74, but did not differ between OVX/E^2^ mice receiving saline or chemotherapy, indicating that this effect was associated with OVX and/or E^2^ supplementation and not a chemotherapy-induced change.

### Test day probe trials

#### General activity

Because the presence and development of tumors, surgical manipulation, and/or the administration of chemotherapeutics could alter activity in the mice and distort or confound the interpretation of spatial memory measures taken on the Day 18, 46, and 74 probe trials, we analyzed the TDM and VEL during the probe trials for differences between groups and changes across days of assessment (Table 2). Results of mixed-factor ANOVA for both TDM and VEL indicated a general tendency for reduced activity across days of assessment (partial *η*^2 ^= 0.481; *F*_(2,64)_ = 29.706; *p* < 0.05). Both TDM and VEL were significantly lower on Day 46 than Day 18 and significantly lower on Day 74 than on either Day 18 or Day 46. Because there were no significant effects of the group or treatment on activity, this effect like reflects a tendency toward reduced activity as the mice mature and have more experience in the MWM, as observed in the Platform Trial 1 data.

**Table 2.** TDM and VEL during probe trials

		Day 18 (baseline)	Day 46 (4 weeks of injections)	Day 74 (5 weeks postinjections)
Sham surgery				
Tap water	TDM (cm)	1,244 ± *36*	1,173 ± *33*	1,008 ± *59*
Saline (*N* = 8)	VEL (cm/s)	20.7 ± *0.6*	19.6 ± *0.6*	16.8 ± *1.0*
Sham surgery				
Tap water	TDM (cm)	1,189 ± *53*	1,076 ± *106*	911 ± *61*
DOX + CYP (*N* = 9)	VEL (cm/s)	19.8 ± *0.9*	17.9 ± *1.8*	15.2 ± *1.0*
OVX				
E^2^ water	TDM (cm)	1,123 ± *50*	976 ± *49*	888 ± *44*
Saline (*N* = 10)	VEL (cm/s)	18.7 ± *0.8*	16.3 ± *0.8*	14.8 ± *0.7*
OVX				
E^2^ Water	TDM (cm)	1,146 ± *44*	1,052 ± *53*	914 ± *84*
DOX + CYP (*N* = 9)	VEL (cm/s)	19.1 ± *0.7*	17.5 ± *0.9*	15.3 ± *1.4*
ALL (*N* = 36)	TDM (cm)	1,176 ± *24*	1,069 *± 34* ↓*	930 *± 32* ↓* ↓↓*
	VEL (cm/s)	19.6 ± *0.4*	17.8 *± 0.6* ↓*	15.5 *± 0.5* ↓* ↓↓*

The activity metrics of sham and OVX mice treated with either saline or a combination of CYP and DOX (CYP + DOX), measured at the baseline (Day 18), 4 weeks post-treatment (Day 46), and 5 weeks after treatment completion (Day 74). Decreases from the baseline are indicated by one downward arrow, while decreases from the post-treatment measurements are denoted by two arrows. All values represent means ± SEM (in italic). All statistical comparisons were made using Sidak's correction for multiple comparisons, with significance (indicated by *) defined at *p* < 0.05. Total sample size (*N*) is 36.

#### Correlations

Prior to analysis of MWM probe trial data via MANOVA, Pearson's *r* partial correlations (Table 3) were calculated between DMCZ, ECZ, TCZ, and LCZ measurements from probe trials conducted on Days 18 (baseline), 46 (after four weekly injections), and 74 (5 weeks following the last injection). These behavioral measures were chosen because they are traditionally considered indicative of knowledge of the platform location during MWM probe trials. Not surprisingly, there were significant correlations between DMCZ, ECZ, TCZ, and LCZ at nearly all timepoints, demonstrating convergent validity and suggesting each measurement was capturing some aspect of a common construct, in this case, spatial memory. However, TCZ was correlated >0.9 with DMCZ indicating these variables did not provide unique information. Therefore, TCZ was not included in the subsequent MANOVA to eliminate the adverse impact of redundancy on the analysis. Additionally, LCZ was negatively correlated with all variables, i.e., poor spatial memory was associated with a longer latency to enter the correct zone for the first time. Therefore, LCZ values from each mouse were inverted by subtracting each latency from the total trial duration (60 s). The inverted values, now positively correlated with the other variables, were used for the subsequent MANOVA. The final values used in the MANOVA were moderately correlated, with Pearson's *r* values between 0.218 and 0.668, indicating that each measure captured unique and overlapping aspects of spatial memory in the MWM.

**Table 3.** Correlation matrices for probe trial metrics at various timepoints

		*DMCZ*	*ECZ*	*TCZ*	*LCZ*
Baseline	*DMCZ*	1	**0.668**	**0**.**897**	**−0**.**312**
	*ECZ*		1	**0**.**506**	**−0**.**471**
	*TCZ*			1	−0.218
	*LCZ*				1
4 weeks injections	*DMCZ*	1	**0.579**	**0**.**926**	**−0**.**391**
	*ECZ*		1	**0**.**408**	**−0**.**346**
	*TCZ*			1	**−0**.**391**
	*LCZ*				1
5 weeks postinjections	*DMCZ*	1	**0.330**	**0**.**871**	**−0**.**623**
	*ECZ*		1	0.226	**−0**.**349**
	*TCZ*			1	**−0**.**620**
	*LCZ*				1

Correlation coefficients between the DMCZ, ECZ, (TCZ, and LCZ across three assessment points: baseline, after 4 weeks of injections, and 5 weeks postinjections. Degrees of freedom (df) = 32. Correlations significant at *p* < 0.05 are highlighted in bold. TCZ was excluded from subsequent MANOVA analyses due to a high correlation coefficient exceeding 0.9, suggesting redundancy. Inverted LCZ values were utilized in the MANOVA to ensure all variables contributed positively to the analysis.

#### Spatial memory

Results of a MANOVA using DMCZ, ECZ, and inverted LCZ measurements from probe trials conducted on Days 18, 46, and 74 demonstrated significant interactions between the group and treatment (partial *η*^2 ^= 0.028; *F*_(3,94)_ = 3.161; *p* < 0.05) and treatment and timepoint (partial *η*^2 ^= 0.065; *F*_(6,188)_ = 2.168; *p* < 0.05) as well as a significant change across Timepoint (partial *η*^2 ^= 0.177; *F*_(6,188)_ = 7.535; *p* < 0.05) suggesting group differences in spatial memory as well as changes in spatial memory across assessment timepoints. Subsequent univariate analyses were conducted on each measure to isolate effects.

#### DMCZ, ECZ, and LCZ analyses (Fig. 4)

*DMCZ*. There were significant effects of timepoint (partial *η*^2 ^= 0.142; *F*_(2,96)_ = 7.915; *p* < 0.05) and an interaction of the group and treatment (partial *η*^2 ^= 0.062; *F*_(1,96)_ = 6.364; *p* < 0.05) on DMCZ.

**Figure 4.**
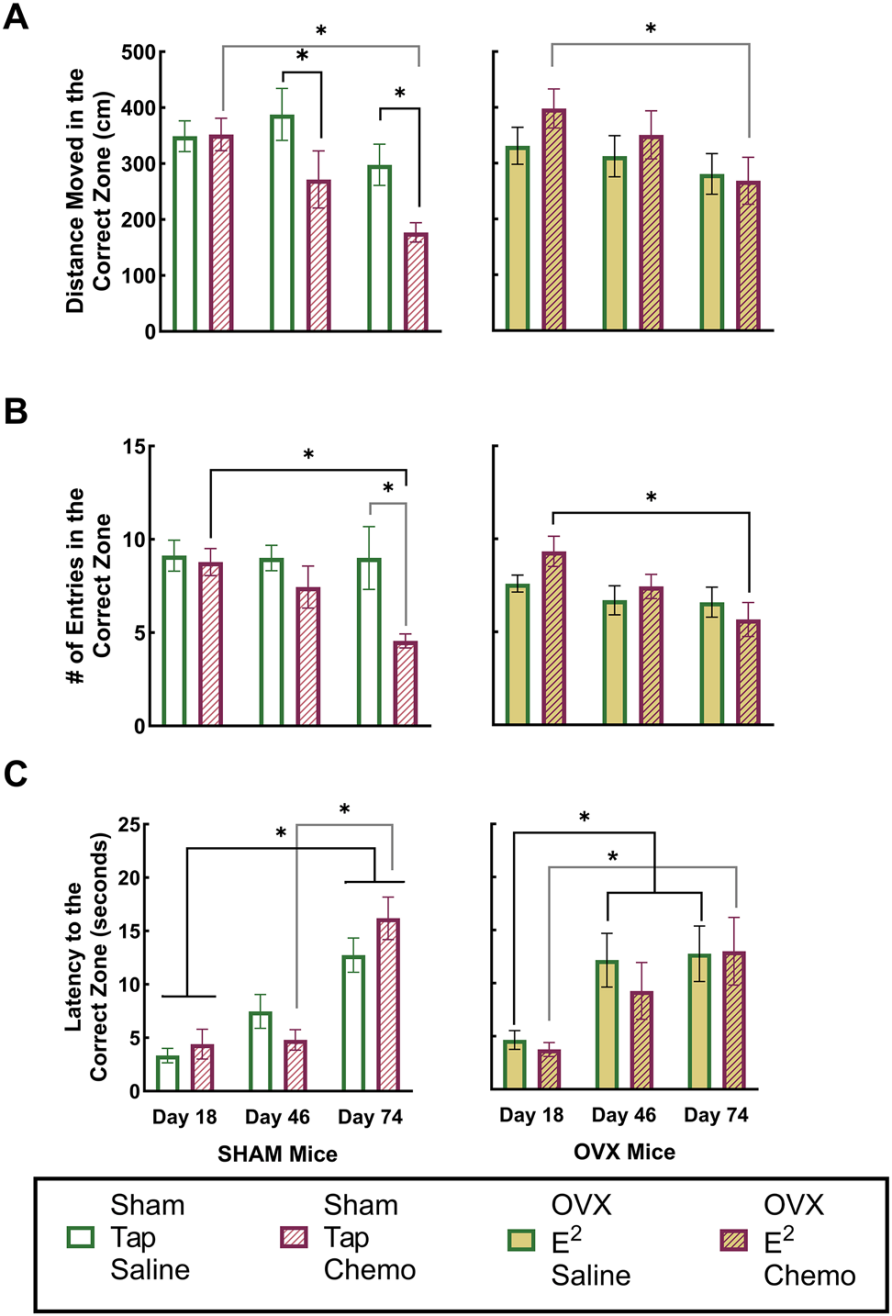
Spatial memory assessment in probe trials. Panels ***A–C*** depict the distance moved within the correct zone (***A***), number of entries to the correct zone (***B***), and latency to reach the correct zone (***C***) across three timepoints: baseline (Day 18), after 4 weeks of either chemotherapy or saline injections (Day 46), and 5 weeks following the conclusion of injections (Day 74). Group means are shown along with SEM. Colors and symbols used to differentiate groups are detailed in the legend. Asterisks highlight statistically significant differences between groups at each timepoint. Significance was determined using a *p* value of <0.05 after applying Sidak's correction for multiple comparisons

Subsequent multiple comparisons using Sidak's correction for family-wise error revealed that sham-operated mice receiving CYP + DOX injections moved significantly less in the correct zone during Day 46 (after four weekly injections) and Day 74 (5 weeks following the final injection) probe trials when compared with sham-operated mice receiving saline injections. Additionally, the DMCZ of sham surgery mice receiving CYP + DOX injections decreased significantly from the baseline (Day 18) to 5 weeks postinjections (Day 74; Fig. 4*A*). These results indicate a CYP + DOX-induced impairment of spatial memory in sham surgery mice receiving chemotherapy. For OVX/E^2^ mice, there was a significant reduction in DMCZ from the baseline (Day 18) to 5 weeks postinjections (Day 74); however, this measure was not significantly lower in CYP + DOX-injected OVX/E^2^ mice than saline-injected OVX/E^2^ mice at any timepoint, indicating this change in the CYP + DOX OVX/E^2^ mice was not due to chemotherapy but a trend in OVX/ E^2^ mice regardless of treatment.

*ECZ (**Fig. 4B**)*. There were significant effects of timepoint (partial *η*^2 ^= 0.126; *F*_(2,96)_ = 6.929; *p* < 0.05), group and treatment (partial *η*^2 ^= 0.069; *F*_(1,96)_ = 7.073; *p* < 0.05), and treatment and timepoint (partial *η*^2 ^= 0.078; *F*_(2,96)_ = 4.057; *p* < 0.05) on ECZ.

Subsequent multiple comparisons using Sidak's correction for family-wise error revealed that sham-operated mice that received CYP + DOX injections entered the correct zone less frequently during Day 74 (5 weeks following the final injection) probe trial than sham-operated mice that received saline injections. Additionally, sham-operated mice that received CYP + DOX injections exhibited a significant decrease in ECZ from Day 18 to Day 74. These findings suggest an impairment of spatial memory in sham surgery mice that emerges several weeks following CYP + DOX exposure. While OVX/E^2^ mice receiving CYP + DOX injections also exhibited a significant decrease in correct zone entries from Day 18 to Day 74, the ECZ of OVX/ E^2^ mice receiving CYP + DOX did not differ significantly from saline-injected OVX/E^2^ mice on Day 74, suggesting the reduction in correct zone entries across weeks of assessment was not due to CYP + DOX administration.

*LCZ (**Fig. 4C**)*. There were significant effects of timepoint (partial *η*^2 ^= 0.333; *F*_(2,96)_ = 23.988; *p* < 0.05) on LCZ.

Subsequent multiple comparisons using Sidak's correction for family-wise error revealed no significant differences between saline and CYP + DOX-injected sham–operated mice at any assessment timepoint. Similarly, for OVX/E^2^ mice, LCZ did not differ between saline and CYP + DOX-injected mice at any timepoint. For sham-operated mice, LCZ did increase significantly on Day 74 (5 weeks following the final injection) compared with either Day 18 (baseline) or Day 46 (following four weekly injections). Similarly, for OVX/E^2^ mice, LCZ increased significantly from Day 18 to Day 74. Taken together these results indicate a tendency for mice to take longer to find the correct zone across weeks of assessment regardless of surgery type or treatment.

**Figure 5. EN-NWR-0206-24F5:**
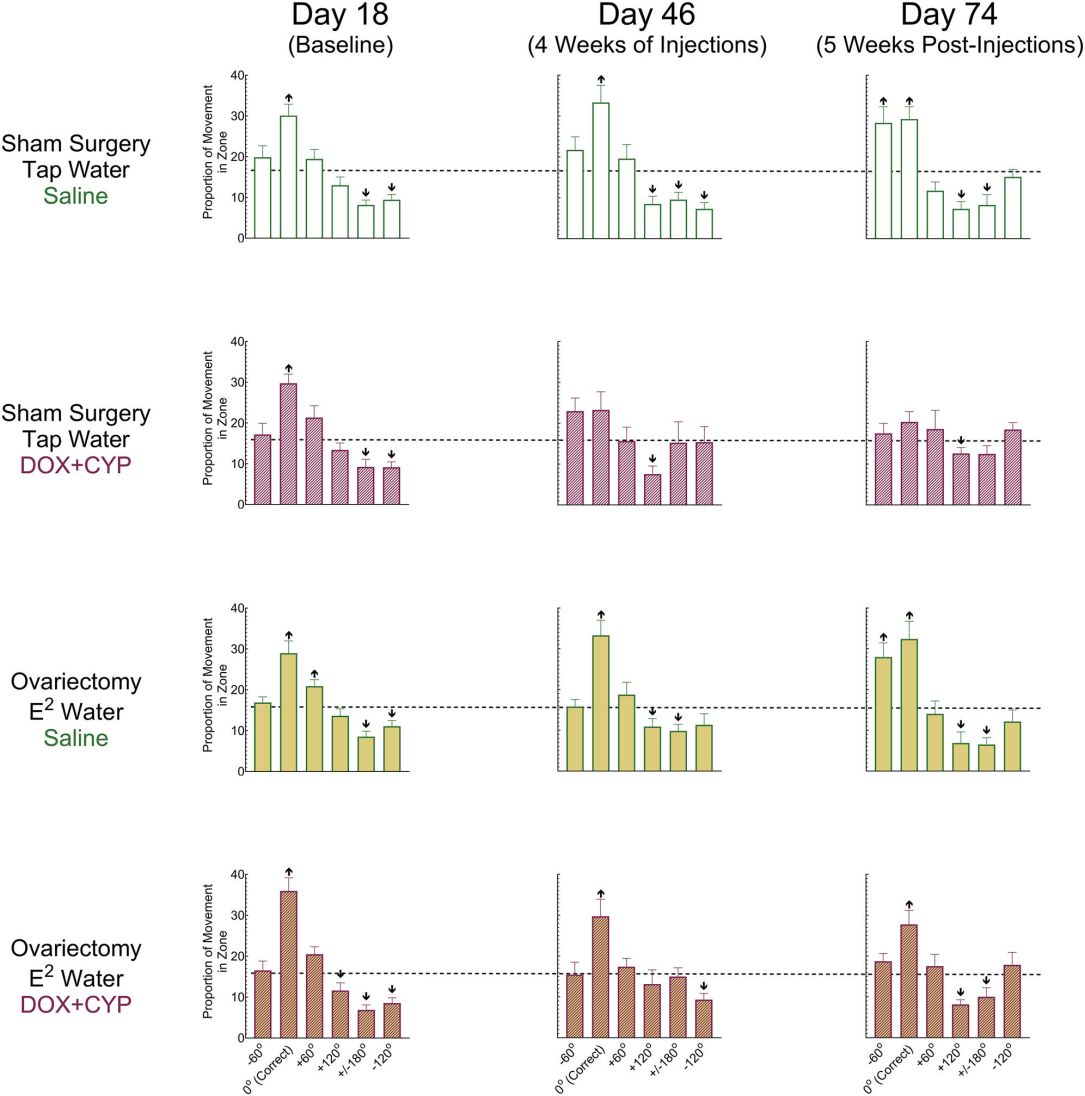
Spatial exploration preferences in MWM zones. The proportion of distance moved within each zone of the MWM for sham and OVX mice at three assessment points: baseline (Day 18), 4 weeks postinjections (Day 46), and 5 weeks after injections (Day 74). Bars denote group means with SEM; the dashed line indicates an unbiased exploration expectation (16.67%). The 0° marks the target zone from the training trials. Each row details one of the four groups defined by OVX status (sham or OVX) and treatment type (saline or CYP + DOX). Upward arrows signify more exploration than unbiased expectation, while downward arrows show less. Statistical significance, marked by Sidak's correction for multiple comparisons, is noted for *p* < 0.05.

*Disproportionate zone exploration*. To confirm the finding that spatial memory was impaired by CYP + DOX in sham surgery mice and that these results were not due to any differences in activity, the proportion of distance moved in each zone during the Day 18, Day 46, and Day 74 probe trials was analyzed (Fig. 5). At the baseline (Day 18), when the proportion of distance moved in each zone was compared with unbiased exploration (100%/6 zones = 16.7% per zone) all groups explored the correct zone (0°) significantly above the expected value (16.67%) for unbiased behavior. Additionally, OVX/E^2^ mice assigned to receive saline injections also explored the +60° zone more than expected for unbiased behavior. In contrast, all groups explored the +/−180° and the −120° zones less than expected for unbiased behavior and OVX/E^2^ mice assigned to receive CYP + DOX injections explored the +120° zone less than expected for unbiased behavior. Taken together, these data indicate that on Day 18 (baseline), all groups could effectively utilize spatial cues to navigate to the correct area of the MWM and were generally aware of areas of the MWM that were not near the correct zone.[Fig EN-NWR-0206-24F5]

On Day 46, following four weekly injections, saline-injected sham/tap mice explored the correct zone (0°) more, and the +120°, +/−180°, and −120° zones less, than expected if the behavior was unbiased. This indicates that the spatial memory of saline-injected sham/tap mice was not impaired by the passage of time or repeated injections. In contrast, for CYP + DOX-injected sham/tap mice, no zones were explored significantly more than expected due to chance during the Day 46 probe trial, indicating an inability to identify the correct zone following four weekly injections of CYP + DOX. These mice did explore the +120° zone significantly less than expected, suggesting some spatial memory; however, the overall distribution of behavior strongly indicates spatial memory impairment.

On Day 46, both saline- and CYP + DOX-injected OVX/E^2^ mice explored the correct zone (0°) more than expected if the behavior was unbiased. Saline-injected OVX/E^2^ mice also explored the +120° and +/−180° zones less than expected and CYP + DOX-injected OVX/E^2^ mice explored the −120° zone less than expected if the behavior were unbiased. Taken together, these data suggest that OVX/E^2^ mice retained spatial memory of the correct zone and that CYP + DOX did not impair spatial memory.

On Day 74, 35 d following the final injection, saline-injected sham/tap mice explored the correct zone (0°) and the −60° zone significantly greater than expected and explored the +120° and +/−180° zones less than expected if the behavior was unbiased. This indicates that saline-injected sham/tap mice retained a spatial memory of the platform location throughout the study. In contrast, CYP + DOX-injected sham/tap mice did not explore any zones more than expected if the behavior was unbiased and explored the −120° zone less than expected if the behavior was unbiased.

This strongly suggests that CYP + DOX injections impair the spatial memory of mice that received sham surgery.

On Day 74, both saline- and CYP + DOX-injected OVX/E^2^ mice explored the correct zone (0°) more than expected if the behavior was unbiased. Saline-injected OVX/E^2^ mice also explored the −60° zone more than expected. Additionally, both saline- and CYP + DOX-injected OVX/E^2^ mice explored the +120° and +/−180° zones less than expected if the behavior was unbiased. Therefore, there does not appear to be a CYP + DOX-induced impairment of spatial memory in OVX/E^2^ mice.

## Discussion

The present study assessed spatial memory in OVX/E^2^ and sham/tap mice on three occasions: before treatment (Day 18), after four weekly injections (Day 46), and 35 d postfinal injection (Day 74) of either saline or CYP + DOX. The study aimed to test the hypothesis that the cognitive impairing effects of AC chemotherapy are dependent on vulnerability of the ovaries to ablation. The results indicate that spatial memory impairment following exposure to CYP + DOX is notably influenced by the status of ovarian function, which affects circulating estrogen levels. Sham-operated mice, experiencing natural ovarian suppression and E^2^ depletion due to chemotherapy, exhibited significant impairments of spatial memory when compared with their saline-injected counterparts and to CYP + DOX-injected OVX mice maintained on estrogen-supplemented water. This finding is important because it demonstrates that even though CYP + DOX was present in the system, unless the ovaries are vulnerable to chemotherapy-induced damage, cognitive deficits do not occur. This suggests that chemotherapy-induced ovarian suppression or ablation heightens vulnerability to CRCIs and underscores the protective role of E^2^ against the effects of chemotherapeutic agents that lead to cognitive decline. These findings contribute to the expanding body of literature on CRCIs, particularly in the context of hormonal influence on neurocognitive outcomes postchemotherapy.

One interpretation of these findings is that reductions in circulating E^2^ due to chemotherapy directly impair cognitive function. It is well established that circulating E^2^ is associated with cognitive performance; for example, E^2^ contributes to normal verbal and spatial memory in females (S. J. Lee and McEwen, 2001; Sherwin, 2003). Furthermore, lower concentrations of circulating E^2^ are linked to poorer performance on cognitive function tests. Specifically, natural reductions in E^2^ during the menstrual cycle are associated with decreased verbal fluency, working memory capacity, perceptual speed, and fine motor function (Hampson, 1990; Phillips and Sherwin, 1992; Rosenberg and Park, 2002). Therefore, it could be argued that the cognitive deficits observed in CRCIs are a direct consequence of lowered E^2^ levels (Sherwin, 2003; Eberling et al., 2004). However, this interpretation does not account for studies of CRCIs that have identified chemotherapy-induced changes in the structure and function of the CNS, suggesting various mechanisms through which CRCIs may manifest (Ahles and Saykin, 2007; Deprez et al., 2011; Simó et al., 2013)

### Estrogens mediate vulnerability to chemotherapeutic agents

Several mechanisms leading to CRCIs have emerged as relevant to the present findings including cytokine-mediated inflammation, microglial activation, oxidative stress, and disruptions in acetylcholine (ACh) synthesis (Berry et al., 2014; Christenson et al., 2017; Fujii et al., 2017, 2015). Each mechanism provides a plausible explanation for how ovarian suppression or ablation, and the consequent reduction in circulating estrogens, may heighten susceptibility to cognitive impairments induced by AC chemotherapy.

Estrogens modulate the immune response within the CNS, particularly by inhibiting the activation of microglia—the primary immune cells in the brain. When microglia are activated, they can exacerbate neuroinflammation, leading to cognitive deficits. By maintaining estrogen levels, either through ovarian function or supplementation, the inflammatory responses triggered by chemotherapy can be significantly reduced, thus preserving cognitive function (Vegeto et al., 2001, 2008; Ghisletti et al., 2005).

Furthermore, estrogens possess antioxidant properties that help in scavenging the free radicals generated during chemotherapy, reducing oxidative stress which is a known contributor to cellular damage and cognitive decline. Estrogens also enhance the expression of antioxidant enzymes, further protecting neural tissues against the oxidative stress induced by both cancer and chemotherapeutic treatments (Razmara et al., 2007).

Studies suggest that E^2^ can modulate the expression and activity of cholinergic systems, which are vital for cognitive processes such as learning and memory (Luine, 2014; Barth et al., 2015). Estradiol has been demonstrated to regulate high-affinity choline uptake (HACU), which is the rate-limiting step in the synthesis of ACh. In mouse models, disruptions of HACU across multiple brain regions have been shown to impact memory and cognition (O’Malley et al., 1987; Ragozzino and Choi, 2004; Bennett et al., 2009; Muramatsu et al., 2016). Thus, chemotherapy-induced reductions in circulating E^2^ can result in CRCIs because of impaired ACh synthesis and cholinergic function. Furthermore, ACh plays a key role in regulating inflammatory responses through the cholinergic anti-inflammatory pathway. Specifically, when ACh binds to α7 nicotinic ACh receptors on macrophages and other immune cells, it leads to the suppression of proinflammatory cytokines by inhibiting the NF-κB pathway, which regulates proinflammatory cytokines such as IL-1β, TNF-α, and IL-6. Consequently, the reduction in HACU induced by chemotherapy, and the subsequent decrease in ACh production, can increase susceptibility to CNS damage caused by neuroinflammation.

### Estrogens mediate sex differences in CRCI vulnerability

The present findings, along with existing literature, highlight the protective effects of circulating E^2^. The impact of chemotherapy on circulating E^2^ suggests why females may be more vulnerable than males to the adverse cognitive effects of chemotherapy. However, males produce E^2^ through the aromatization of testosterone from the testes, with circulating concentrations ranging from 10 to 40 pg/ml. For comparison, adult premenopausal females have circulating E^2^ concentrations from 15 to 350 pg/ml, while postmenopausal females exhibit circulating E^2^ concentrations ∼10 pg/ml [111]. Because chemotherapy can lead to gonadal suppression or ablation in both sexes, males could also see reductions in circulating E^2^ during and after treatment, potentially increasing their vulnerability to these agents.

One factor contributing to the greater vulnerability of females to the negative effects of chemotherapy is the rapid division of cells in the ovarian follicles, which house and support the oocytes and are the primary source of circulating E^2^. The follicular cells are highly susceptible to damage from chemotherapeutic agents, which preferentially target rapidly dividing cells. This damage can disrupt the support system for the oocytes, leading to their dysfunction or death. Importantly, since females are born with a finite number of oocytes, any damage to these cells is irreversible and has long-term consequences for hormonal balance.

In contrast, Leydig cells in the testes produce testosterone and do not divide rapidly. Thus, these cells are less vulnerable to chemotherapies targeting rapidly dividing cells. Additionally, Leydig cells are relatively isolated from direct contact with blood-borne chemotherapeutic agents compared with ovarian follicles. These cells also typically operate below their maximal synthetic capacity, which allows them to maintain sufficient testosterone production even if some cells are damaged. Moreover, the testes contain progenitor Leydig cells capable of differentiating and expanding in response to injury or loss. Although this regenerative potential is limited, it offers a means to restore function over time if the initial damage is not overwhelming.

Taken together, this evidence suggests that the testes would be less vulnerable to systemic chemotherapy than the ovaries, and therefore, circulating E^2^ in males is less likely to be impaired by chemotherapy than in females.

### Estrogen supplementation

The current results suggest that maintaining physiological levels of estrogen postchemotherapy could be beneficial in preserving cognitive function, opening the door to targeted therapeutic strategies that consider hormonal status as a factor in treatment planning. However, this potential protection comes with a caveat: direct hormonal replacement therapy has proven to be problematic. ER-positive tumors are associated with as high as double the risk of recurrence and metastasis (Dorgan et al., 2010), especially following menopause. Estradiol can induce direct DNA changes complicating DNA repair (Haakensen et al., 2011; Li et al., 2018; J. J. K. Lee et al., 2023) and enhance cellular proliferation and tumor invasiveness in ER-positive breast cancer cells (Haakensen et al., 2011; T. Wang et al., 2021; Pacheco-Velázquez et al., 2022; Gnant et al., 2023) by activating pathways such as ERK/MAPK and Wnt/β-catenin, which are crucial for cell proliferation and differentiation in cancer cells. Therefore, estrogen supplementation during chemotherapy is not advisable for ER-positive tumors as E^2^ is known to induce metastasis, proliferation, differentiation, and recurrence of cancer cells (Chen et al., 2020). Indeed, the tumor volumes in our OVX/E^2^ mice were consistently larger than their sham/tap counterparts during the last few weeks of assessment.

### Conclusions

This study effectively demonstrates that E^2^ acts as a protective agent against the cognitive impairments associated with AC chemotherapy, specifically in the context of ovarian function and its consequential impact on circulating estrogen. Sham-operated mice, which experienced natural ovarian suppression and subsequent declines in circulating E^2^, exhibited significant deficits in spatial memory following chemotherapy, while OVX mice maintained on E^2^-supplemented water did not exhibit chemotherapy-induced impairment of spatial memory. This protective effect highlights the critical role of E^2^ in buffering the neurotoxic effects of chemotherapeutic agents that are likely exacerbated by induced ovarian suppression. This underscores the importance of considering hormonal health in the treatment of female cancer patients and may advocate for strategies that support estrogen levels during chemotherapy to mitigate cognitive decline in the absence of ER-positive tumors.

## Synthesis

Reviewing Editor: Jibran Khokhar, Western University Department of Anatomy and Cell Biology Decisions are customarily a result of the Reviewing Editor and the peer reviewers coming together and discussing their recommendations until a consensus is reached. When revisions are invited, a fact-based synthesis statement explaining their decision and outlining what is needed to prepare a revision will be listed below. The following reviewer(s) agreed to reveal their identity: Melanie Sekeres, Jill Daniel.

The authors assessed the impact of chemotherapy-related cognitive impairment (CRCI) on spatial memory in the transgenic MMTV-PyVT tumour-bearing mouse model, and the role of estrogen in protecting against CRCI. They found that combination DOX+Cyclo chemotherapy-induced ovarian suppression or ablation was associated with spatial memory deficits in the Morris Water Maze (MWM). Maintaining physiological levels of estrogen via E2 supplement in OVX-mice post-chemotherapy protected against these deficits, suggesting that chemotherapy-induced estrogen suppression may be a leading mechanism responsible for the high rates of CRCI in females. This well-written and interesting study aims to understand the implications for spatial memory of ovarian depletion induced by chemotherapy using the MMTV-PyVT mouse model. The question is important, and the Introduction and Discussion are appropriate. However, there are significant limitations due to the cumulative development of tumors throughout the longitudinal study and the potential confounding impact of high tumor load on interpreting motor/cognitive behavior.

A few points raised by the reviewers have been synthesized below and will help to improve the clarity and impact of the manuscript:

Measurement Approach: The reliance exclusively on probe trial data in the MWM task limits interpretation. Details on the reassessment on days 46 and 74 are missing. Information about whether the platform location remained the same and if the animals were retrained is crucial as different procedures assess different types of memory (working vs. reference; long-term vs. short-term).

Data on Platform Finding: No data are provided regarding the ability of animals to find the platform on test days. The first trial with the platform in the maze on reassessment days could provide significant information about long-term memory. How quickly the animals relearn the location compared to day 18 would also be informative.

Rationale and Methods: The rationale for using a 50mm3 tumor size as Day 1 for each mouse needs clarification. Additionally, the dosages and number of DOX and CYP injections should be explained.

Visual Acuity: Given the MMTV-PyVT mouse model's susceptibility to retinal problems, it is important to know how visual acuity was confirmed. Information on whether any mice failed to locate the visible platform during MWM training should be included.

Motor Performance and Tumor Load: High tumor load could impact motor performance in the MWM task, confounding results. The authors should present mean overall distance traveled and mean speed for each group in the MWM to ensure motor performance was not the cause of observed deficits.

Body Condition and Attrition: Details on body condition monitoring, endpoint criteria, sickness behavior, weight loss, and attrition during the experiment are necessary.

Statistical Analysis: The range of "moderately correlated measures (r=0.2-0.7)" for the MWM MANOVA analyses should be justified. Also, providing exact p-values and considering scatterplots for individual subject scores in figures would enhance clarity.

Minor Corrections: Define E2 the first time it is used, correct the F value on line 327, and specify the software used for statistical analyses. The discussion on cellular mechanisms of CRCI not assessed in the study could be minimized to avoid an extensive literature review.

## Author Response

Jibran Khokhar, PhD Reviewing Editor eNeuro Dear Dr. Khokhar, Attached is a revision of a manuscript eN-NWR-0206-24 for reconsideration for publication in eNeuro. We thank the Editors for handling the review of this manuscript and the thoughtful consideration by the reviewers. We feel that the new version of the manuscript is greatly improved because of the reviewer's suggestions. The reviewer's concerns and how they have been addressed are detailed below using hanging indents:

Synthesis of Reviews:

Computational Neuroscience Model Code Accessibility Comments for Author (Required): N/A Synthesis Statement for Author (Required):

The authors assessed the impact of chemotherapy-related cognitive impairment (CRCI) on spatial memory in the transgenic MMTV-PyVT tumour-bearing mouse model, and the role of estrogen in protecting against CRCI. They found that combination DOX+Cyclo chemotherapy-induced ovarian suppression or ablation was associated with spatial memory deficits in the Morris Water Maze (MWM). Maintaining physiological levels of estrogen via E2 supplement in OVX-mice post-chemotherapy protected against these deficits, suggesting that chemotherapy-induced estrogen suppression may be a leading mechanism responsible for the high rates of CRCI in females. This well-written and interesting study aims to understand the implications for spatial memory of ovarian depletion induced by chemotherapy using the MMTV-PyVT mouse model. The question is important, and the Introduction and Discussion are appropriate. However, there are significant limitations due to the cumulative development of tumors throughout the longitudinal study and the potential confounding impact of high tumor load on interpreting motor/cognitive behavior.

A few points raised by the reviewers have been synthesized below and will help to improve the clarity and impact of the manuscript:

1. Measurement Approach: a. The reliance exclusively on probe trial data in the MWM task limits interpretation.

At the suggestion of the reviewers, we have included some analyses of the platform trial data. However, we believe that emphasizing platform trials would confuse interpretation and understanding for multiple reasons. Platform trials cannot be relied upon as an indication of spatial memory. Platform trials can indicate that an effective search strategy had been developed for when the platform is present, but not whether a memory of the platform location based on spatial cues is present.

Additionally, platform trials cannot be used as an indicator of general activity because trial duration is not constant, ending when the animal locates the platform, and thus each animals' activity measures will be distorted by time. This is true of swim velocity as well because the initial response to being placed in the water varies for each animal (activation in some, passivity in others) and changes over the duration of the trial. b. Details on the reassessment on days 46 and 74 are missing. Information about whether the platform location remained the same and if the animals were retrained is crucial as different procedures assess different types of memory (working vs. reference; long-term vs. short-term).

We have updated the methods section to clarify that the platform location is constant for each animal for the duration of the study and added details to the 'Reassessment of Spatial Memory' section to clearly describe the reassessment process and purpose. Retraining mice to a new platform location does not occur in these studies.

2. Data on Platform Finding: a. No data are provided regarding the ability of animals to find the platform on test days. The first trial with the platform in the maze on reassessment days could provide significant information about long-term memory.

We agree that the first platform trial on Days 46 and 74 could indicate the stability of some forms of long-term memory and have included an analysis, discussion and a new figure of group differences during these trials at the suggestion of the reviewers. b. How quickly the animals relearn the location compared to day 18 would also be informative.

We have revised the methods section to include more details about MWM training and reassessments. For training animals received to 5 trials per day for 5 consecutive days prior to baseline assessment on day 18. Mice were not 'retrained' to a platform location on days 46 or 74 in any comparable way. Mice receive 2 platform trials prior to the spatial memory probe trial on days 46 and 74, thus the ability to make determinations about 'retraining' from the data would not be reasonable as we would only be able to measure the change from trial 1 to trial 2 which is not comparable to 5 trials/day for 5 days.

3. Rationale and Methods: a. The rationale for using a 50mm3 tumor size as Day 1 for each mouse needs clarification.

We have added the rationale for using a 50mm3 tumor size as the study starting point to the Methods section. b. Additionally, the dosages and number of DOX and CYP injections should be explained.

We have added the rationale for the treatment regimen used to the 'Chemicals and Injections' section of the Methods.

4. Visual Acuity: a. Given the MMTV-PyVT mouse model's susceptibility to retinal problems, it is important to know how visual acuity was confirmed.

We have modified the methods section to clarify that a crucial reason for choosing B6.FVB-Tg (MMTV-PyVT) 634Mul/LellJ mice rather than FVB-TgN(MMTV-PyVT) mice is because the former does not exhibit the vulnerability to retinal degeneration and reduction of visual acuity that is present in the latter. We apologize for not including this detail in the prior version and appreciate this important observation. We did not specifically test visual acuity in these mice. b. Information on whether any mice failed to locate the visible platform during MWM training should be included.

No mice failed to locate the visible platform during orientation. The platform was not visible during training. We have added to the methods section the number of mice that when tested at baseline failed to achieve the minimum criteria of 20% of movement in the correct zone, indicating they did not have a spatial memory of the platform location.

5. Motor Performance and Tumor Load: a. High tumor load could impact motor performance in the MWM task, confounding results. The authors should present mean overall distance traveled and mean speed for each group in the MWM to ensure motor performance was not the cause of observed deficits.

We have added an analysis of the total distance moved and swim velocity of mice during the probe trials and a summary table of the group means and errors. There were no differences between groups on the measures but there was a general tendency for a reduction in activity across days of assessment regardless of group assignment. This agrees with the platform trial analysis we have also include as requested and indicates that group differences in activity due to tumor burden or other possible factors are unlikely to confound the reported results.

It is important to note that the primary purpose for presenting the probe trial data as the proportion of movement devoted to each zone is to control for differences in motor performance that could occur due to tumor load or the effects of chemotherapy. The distribution of behavior across all zones does not conceal from the reader bias present in the mice and we feel is the best way to objectively represent spatial memory when differences in activity are a concern.

6. Body Condition and Attrition: a. Details on body condition monitoring, endpoint criteria, sickness behavior, weight loss, and attrition during the experiment are necessary.

The initial number of mice, reason for removal from the analysis and final N have been added to the methods. Details of body condition monitoring and endpoint criteria have been added to the methods in a section titled "Attrition." 7. Statistical Analysis: a. The range of "moderately correlated measures (r=0.2-0.7)" for the MWM MANOVA analyses should be justified.

The rationale has been added to the "Statistical Analysis" section of the methods. b. Also, providing exact p-values and considering scatterplots for individual subject scores in figures would enhance clarity.

As acknowledged in the eNeuro guidelines, p-values from traditional hypothesis testing are often misinterpreted as indicators of effect magnitude, contributing to the journal's request to perform equivalence testing where applicable. However, defining clinically relevant thresholds for practical significance is not appropriate for assessing behavioral changes in mice using the Morris water maze. Therefore, this study employs traditional hypothesis testing with a conventional significance threshold of 0.05 to determine if group differences are unlikely to result from chance.

Reporting specific p-values can contribute to the misconception that the size of these values indicate effect magnitude, which is not appropriate. Instead, we report results as either exceeding or failing to exceed the conventional significance threshold, aligning with a Yes/No decision approach. It is important to note that p-values do not indicate practical significance. To assist in assessing practical significance, we have included traditional measures of effect size in our analyses. This approach ensures that our findings are based on unbiased criteria and provides a clearer understanding of the practical implications of our results.

Our analyses compared multiple conditions across various timepoints and zones. Scatterplots do not effectively highlight the differences and similarities between these groups. To facilitate easier comparisons, we have chosen to use bar charts with error bars. This approach clearly shows the central tendency and variability within each group, which are critical components underlying the statistical analyses performed and presented in the text of the results.

8. Minor Corrections: a. Define E2 the first time it is used, correct the F value on line 327, and specify the software used for statistical analyses.

E2 is now defined the first time it is used in the introduction. The typo in the F value has been corrected. Thank you for catching these errors. Additionally, we have added the software used for statistical analysis to the methods section. b. The discussion on cellular mechanisms of CRCI not assessed in the study could be minimized to avoid an extensive literature review.

The discussion section has been revised and the discussion of possible cellular mechanisms has been minimized.
